# Supplementary material for: Decision impact studies, evidence of clinical utility for genomic assays in cancer: A scoping review
Source: PLoS One. 2023 Mar 10;18(3):e0280582. doi: 10.1371/journal.pone.0280582 (PMC10004522; doi:10.1371/journal.pone.0280582)
Supplement: S2 Appendix — (DOCX) [file pone.0280582.s002.docx]

**S2 Appendix. Full electronic search strategy for Scopus database**

**Scopus Search Strategy:**

ALL("decision impact" or "decision-impact" or "decision-making impact" or "decision making impact") AND ( EXCLUDE ( DOCTYPE,"ch" ) OR EXCLUDE ( DOCTYPE,"bk" ) OR EXCLUDE ( DOCTYPE,"no" ) OR EXCLUDE ( DOCTYPE,"ed" ) OR EXCLUDE ( DOCTYPE,"le" ) OR EXCLUDE ( DOCTYPE,"sh" ) ) AND ( EXCLUDE ( LANGUAGE,"Spanish" ) OR EXCLUDE ( LANGUAGE,"French" ) OR EXCLUDE ( LANGUAGE,"German" ) OR EXCLUDE ( LANGUAGE,"Portuguese" ) OR EXCLUDE ( LANGUAGE,"Chinese" ) OR EXCLUDE ( LANGUAGE,"Japanese" ) OR EXCLUDE ( LANGUAGE,"Persian" ) OR EXCLUDE ( LANGUAGE,"Italian" ) OR EXCLUDE ( LANGUAGE,"Czech" ) )
