# Supplementary material for: Decision impact studies, evidence of clinical utility for genomic assays in cancer: A scoping review
Source: PLoS One. 2023 Mar 10;18(3):e0280582. doi: 10.1371/journal.pone.0280582 (PMC10004522; doi:10.1371/journal.pone.0280582)
Supplement: S1 Table — (DOCX) [file pone.0280582.s003.docx]

**S1 Table. Table of included studies**

| **No.** | **Authors, Year** | **Title** | **Geographic Location (Country)** | **Study Design** | **Type of Cancer Assessed** | **Assay Studied*** | **Reported Outcomes Against FT Model Levels** |
| --- | --- | --- | --- | --- | --- | --- | --- |
| 1 | Akerley et al., 2013 | The impact of a serum based proteomic mass spectrometry test on treatment recommendations in advanced non-small-cell lung cancer | USA | Prospective | Lung | VeriStrat (Biodesix) | L3—Diagnostic thinking efficacy |
| 2 | Antoine et al., 2018 | Paradygm: Impact of 21 Genes Recurrence Score Assay (ODX) on final decision and heterogeneity of decisions between different tumor boards | France | Retrospective | Breast | Oncotype Dx (Genomic Health/Exact Sciences) | L3—Diagnostic thinking efficacy  L4—Therapeutic efficacy |
| 3 | Badani et al., 2015 | Effect of a genomic classifier test on clinical practice decisions for patients with high-risk prostate cancer after surgery | USA | Prospective | Prostate | Decipher (Veracyte) | L3—Diagnostic thinking efficacy |
| 4 | Badani et al., 2013 | Impact of a genomic classifier of metastatic risk on postoperative treatment recommendations for prostate cancer patients: A report from the DECIDE study group | USA | Prospective | Prostate | Decipher (Veracyte) | L3—Diagnostic thinking efficacy |
| 5 | Bargallo-Rocha et al., 2015 | A study of the impact of the 21-gene breast cancer assay on the use of adjuvant chemotherapy in women with breast cancer in a Mexican public hospital | Mexico | Prospective | Breast | Oncotype Dx (Genomic Health/Exact Sciences) | L3—Diagnostic thinking efficacy |
| 6 | Bargallo-Rocha et al., 2012 | A study of the impact of the 21-gene breast cancer assay on the use of adjuvant chemotherapy in women with breast cancer in a Mexican public hospital | Mexico | Prospective | Breast | Oncotype Dx (Genomic Health/Exact Sciences) | L3—Diagnostic thinking efficacy |
| 7 | Barni et al., 2022 | Is the oncotype DX test useful in elderly breast cancer patients: a subgroup analysis of real-life Italian PONDx study | Italy | Prospective | Breast | Oncotype Dx (Genomic Health/Exact Sciences) | L3—Diagnostic thinking efficacy  L4—Therapeutic efficacy |
| 8 | Barni et al., 2020 | Value of genomic test (Oncotype DX) in elderly patients: An Italian survey | Italy | Prospective | Breast | Oncotype Dx (Genomic Health/Exact Sciences) | L3—Diagnostic thinking efficacy  L4—Therapeutic efficacy |
| 9 | Barry et al., 2022 | A prospective decision impact study to evaluate the utility of the Oncotype DX Breast DCIS score assay in selecting patients with ductal carcinoma in situ (DCIS) following breast conservation surgery (BCS) for radiotherapy. | Ireland | Prospective | Breast | Oncotype Dx (Genomic Health/Exact Sciences) | L3—Diagnostic thinking efficacy |
| 10 | Blohmer et al., 2011 | German multicentre decision impact study of Oncotype DX recurrence score (RS) on adjuvant treatment in estrogen receptor positive (ER plus) node negative (N0) and node positive (N plus) early breast cancer | Germany | Prospective | Breast | Oncotype Dx (Genomic Health/Exact Sciences) | L3—Diagnostic thinking efficacy  L4—Therapeutic efficacy |
| **No.** | **Authors, Year** | **Title** | **Geographic Location (Country)** | **Study Design** | **Type of Cancer Assessed** | **Assay Studied** | **Reported Outcomes Against FT Model Levels** |
| 11 | Blumenthal et al., 2016 | Clinical utility and treatment outcome of comprehensive genomic profiling in high grade glioma patients | Israel | Prospective | High grade glioma | FoundationOne (Foundation Medicine) | L3—Diagnostic thinking efficacy  L4—Therapeutic efficacy  L5—Patient outcome efficacy |
| 12 | Brenner et al., 2016 | Impact of the 12-gene colon cancer assay on clinical decision making for adjuvant therapy in stage II colon cancer patients | Israel | Retrospective | Colon | Oncotype Dx (Genomic Health/Exact Sciences) | L3—Diagnostic thinking efficacy |
| 13 | Cheung et al., 2014 | Initial experience with the Oncotype Dx Assay in decision-making for adjuvant therapy of early oestrogen receptor-positive breast cancer in Hong Kong | Hong Kong | Retrospective | Breast | Oncotype Dx (Genomic Health/Exact Sciences) | L3—Diagnostic thinking efficacy |
| 14 | Chin-Lenn et al., 2018 | The impact and indications for Oncotype DX on adjuvant treatment recommendations when third-party funding is unavailable | Australia | Retrospective | Breast | Oncotype Dx (Genomic Health/Exact Sciences) | L3—Diagnostic thinking efficacy |
| 15 | Chin-Lenn et al., 2016 | Indications for, and impact of oncotype DX on adjuvant treatment recommendations when third party funding is unavailable | Australia | Retrospective | Breast | Oncotype Dx (Genomic Health/Exact Sciences) | L3—Diagnostic thinking efficacy |
| 16 | Cognetti et al., 2021 | PONDx: real-life utilization and decision impact of the 21-gene assay on clinical practice in Italy | Italy | Prospective | Breast | Oncotype Dx (Genomic Health/Exact Sciences) | L3—Diagnostic thinking efficacy |
| 17 | Coquerelle et al., 2020 | Impact of Next Generation Sequencing on Clinical Practice in Oncology in France: Better Genetic Profiles for Patients Improve Access to Experimental Treatments | France | Retrospective | Lung, Colorectal, Melanoma | ‘next generation sequencing’ (non-specific) | L3—Diagnostic thinking efficacy  L5—Patient outcome efficacy |
| 18 | Curtit et al., 2019 | Results of PONDx, a prospective multicenter study of the Oncotype DX (R) breast cancer assay: Real-life utilization and decision impact in French clinical practice | France | Prospective | Breast | Oncotype Dx (Genomic Health/Exact Sciences) | L3—Diagnostic thinking efficacy  L4—Therapeutic efficacy |
| 19 | De Boer et al., 2013 | The impact of a genomic assay (Oncotype Dx) on adjuvant treatment recommendations in early breast cancer | Australia | Prospective | Breast | Oncotype Dx (Genomic Health/Exact Sciences) | L3—Diagnostic thinking efficacy  L4—Therapeutic efficacy |
| 20 | De Boer et al., 2011 | Australian decision impact study: The impact of Oncotype DX recurrence score (RS) on adjuvant treatment decisions in hormone receptor positive (HR+), node negative (N0) and node positive (N+) early stage breast cancer (ESBC) in the multidisciplinary clinic (MDC) | Australia | Prospective | Breast | Oncotype Dx (Genomic Health/Exact Sciences) | L3—Diagnostic thinking efficacy  L4—Therapeutic efficacy |
| **No.** | **Authors, Year** | **Title** | **Geographic Location (Country)** | **Study Design** | **Type of Cancer Assessed** | **Assay Studied*** | **Reported Outcomes Against FT Model Levels** |
| 21 | Degtiar et al., 2013 | A prospective registry study assessing decision impact and patient outcomes following gene-expression profiling for tumor-site origin | USA | Prospective | Unknown cancer | Tissue of Origin Test (Pathwork Diagnostics) | L3—Diagnostic thinking efficacy  L4—Therapeutic efficacy  L5—Patient outcome efficacy |
| 22 | Dieci et al., 2019 | Impact of 21-Gene Breast Cancer Assay on Treatment Decision for Patients with T1T3, N0 N1, Estrogen Receptor-Positive/Human Epidermal Growth Receptor 2-Negative Breast Cancer: Final Results of the Prospective Multicenter ROXANE Study | Italy | Prospective | Breast | Oncotype Dx (Genomic Health/Exact Sciences) | L3—Diagnostic thinking efficacy  L4—Therapeutic efficacy |
| 23 | Eiermann et al., 2013 | The 21-gene recurrence score assay impacts adjuvant therapy recommendations for er-positive, node negative and node-positive early breast cancer resulting in a risk-adapted change in chemotherapy use | Germany | Prospective | Breast | Oncotype Dx (Genomic Health/Exact Sciences) | L3—Diagnostic thinking efficacy  L4—Therapeutic efficacy  L5—Patient outcome efficacy  L6—Societal outcome efficacy |
| 24 | Epelbaum et al., 2015 | Molecular Profiling-Selected Therapy for Treatment of Advanced Pancreaticobiliary Cancer: A Retrospective Multicenter Study | Israel | Retrospective | Pancreaticobiliary | Target Now (Caris Life Sciences) | L3—Diagnostic thinking efficacy  L4—Therapeutic efficacy  L5—Patient outcome efficacy |
| 25 | Epelbaum et al., 2013 | Molecular profiling (MP)-selected therapy for the treatment of patients with advanced pancreaticobiliary cancer (PBC) | Israel | Retrospective | Pancreaticobiliary | Target Now (Caris Life Sciences) | L3—Diagnostic thinking efficacy  L4—Therapeutic efficacy  L5—Patient outcome efficacy |
| 26 | Esin et al., 2019 | Prosigna assay for treatment decisions in early breast cancer: A single center, decision impact study | Turkey | Prospective | Breast | Prosigna (Veracyte) | L3—Diagnostic thinking efficacy |
| 27 | Ettl et al., 2017 | Decision impact and feasibility of different ASCO-recommended biomarkers in early breast cancer: Prospective comparison of molecular marker EndoPredict and protein marker uPA/PAI-1 | Germany | Prospective | Breast | Endopredict (Myriad Genetics) | L3—Diagnostic thinking efficacy |
| 28 | Fallowfield et al., 2018 | Enhancing decision-making about adjuvant chemotherapy in early breast cancer following EndoPredict testing | UK | Prospective | Breast | Endopredict (Myriad Genetics) | L3—Diagnostic thinking efficacy  L5—Patient outcome efficacy |
| 29 | Ferguson et al., 2016 | Impact of a bronchial genomic classifier on clinical decision making in patients undergoing diagnostic evaluation for lung cancer | USA | Prospective | Lung | Percepta (Veracyte) | L3—Diagnostic thinking efficacy |
| 30 | Gligorov et al., 2017 | The 21-gene assay in the decision impact assessment of ER+, HER2- Breast cancer: A French real life prospective study | France | Prospective | Breast | Oncotype Dx (Genomic Health/Exact Sciences) | L3—Diagnostic thinking efficacy  L6—Societal outcome efficacy |
| **No.** | **Authors, Year** | **Title** | **Geographic Location (Country)** | **Study Design** | **Type of Cancer Assessed** | **Assay Studied*** | **Reported Outcomes Against FT Model Levels** |
| 31 | Gligorov et al., 2015 | Prospective clinical utility study of the use of the 21-gene assay in adjuvant clinical decision making in women with estrogen receptor-positive early invasive breast cancer: Results from the SWITCH study | France | Prospective | Breast | Oncotype Dx (Genomic Health/Exact Sciences) | L3—Diagnostic thinking efficacy |
| 32 | Gomez et al., 2021 | Practice-changing use of the 21-Gene test for the management of patients with early-stage breast cancer in Latin America | Argentina, Columbia, Mexico, Peru | Prospective | Breast | Oncotype Dx (Genomic Health/Exact Sciences) | L3—Diagnostic thinking efficacy |
| 33 | Hay et al., 2020 | Identifying Opportunities and Challenges for Patients with Sarcoma as a Result of Comprehensive Genomic Profiling of Sarcoma Specimens | USA | Prospective | Sarcoma | FoundationOne (Foundation Medicine) | L3—Diagnostic thinking efficacy  L4—Therapeutic efficacy |
| 34 | Hequet et al., 2021 | Prosigna test in breast cancer: real-life experience | France | Retrospective | Breast | Prosigna (Veracyte) | L3—Diagnostic thinking efficacy  L4—Therapeutic efficacy  L6—Societal outcome efficacy |
| 35 | Hequet et al., 2017 | Prospective, multicenter French study evaluating the clinical impact of the Breast Cancer Intrinsic Subtype-Prosigna (R) Test in the management of early-stage breast cancers | France | Prospective | Breast | Prosigna (Veracyte) | L3—Diagnostic thinking efficacy  L5—Patient outcome efficacy |
| 36 | Hequet et al., 2020 | Prosigna test in clinical routine: Impact on adjuvant chemotherapy decision and medicoeconomic considerations in France | France | Prospective | Breast | Prosigna (Veracyte) | L3—Diagnostic thinking efficacy  L4—Therapeutic efficacy  L6—Societal outcome efficacy |
| 37 | Hogarth et al., 2016 | The percepta registry: A prospective registry to evaluate percepta bronchial genomic classifier patient data | USA | Prospective | Lung | Percepta (Veracyte) | L3—Diagnostic thinking efficacy |
| 38 | Holt et al., 2013 | A decision impact, decision conflict and economic assessment of routine Oncotype DX testing of 146 women with node-negative or pNImi, ER-positive breast cancer in the U.K. | Wales | Prospective | Breast | Oncotype Dx (Genomic Health/Exact Sciences) | L3—Diagnostic thinking efficacy  L5—Patient outcome efficacy  L6—Societal outcome efficacy |
| 39 | Jaafar et al., 2014 | Impact of Oncotype DX testing on adjuvant treatment decisions in patients with early breast cancer: A single-center study in the United Arab Emirates | UAE | Retrospective | Breast | Oncotype Dx (Genomic Health/Exact Sciences) | L3—Diagnostic thinking efficacy  L4—Therapeutic efficacy |
| 40 | Kuchel et al., 2016 | The impact of the 21-gene assay on adjuvant treatment decisions in oestrogen receptor-positive early breast cancer: A prospective study | UK | Prospective & Retrospective | Breast | Oncotype Dx (Genomic Health/Exact Sciences) | L3—Diagnostic thinking efficacy  L5—Patient outcome efficacy |
| **No.** | **Authors, Year** | **Title** | **Geographic Location (Country)** | **Study Design** | **Type of Cancer Assessed** | **Assay Studied*** | **Reported Outcomes Against FT Model Levels** |
| 41 | Kummel et al., 2012 | The Oncotype DX Recurrence Score Assay impacts adjuvant therapy recommendations for ER-positive (ER+), node negative (N0) and node positive (N+) early breast cancer-final results of the German decision impact study | Germany | Prospective | Breast | Oncotype Dx (Genomic Health/Exact Sciences) | L3—Diagnostic thinking efficacy  L4—Therapeutic efficacy  L5—Patient outcome efficacy  L6—Societal outcome efficacy |
| 42 | LeVasseur et al., 2022 | Impact of the 21-Gene Recurrence Score Assay on the Treatment of Estrogen Receptor-Positive, HER2-Negative, Breast Cancer Patients With 1-3 Positive Nodes: A Prospective Clinical Utility Study. | Canada | Prospective | Breast | Oncotype Dx (Genomic Health/Exact Sciences) | L3—Diagnostic thinking efficacy |
| 43 | Martin et al., 2015 | Prospective study of the impact of the ProsignaTM assay on adjuvant clinical decision-making in women with estrogen receptor-positive, HER2-negative, node-negative breast cancer: A GEICAM study | Spain | Prospective | Breast | Prosigna (Veracyte) | L3—Diagnostic thinking efficacy  L4—Therapeutic efficacy |
| 44 | Mattar et al., 2021 | Substantial Reduction in Adjuvant Chemotherapy with the Use of the 21-Gene Test to Manage Early Breast Cancer in a Public Hospital in Brazil | Brazil | Prospective | Breast | Oncotype Dx (Genomic Health/Exact Sciences) | L3—Diagnostic thinking efficacy |
| 45 | McKiernan et al., 2018 | Development of a clinical implementation plan (CarePath) for a novel urine exosome gene expression assay as part of a two-cohort, adaptive decision impact utility trial | USA | Prospective | Prostate | ExoDx Prostate (Exosome Diagnostics) | L3—Diagnostic thinking efficacy |
| 46 | McSorley et al., 2021 | Real-world analysis of clinical and economic impact of 21-gene recurrence score (RS) testing in early-stage breast cancer (ESBC) in Ireland | Ireland | Retrospective | Breast | Oncotype Dx (Genomic Health/Exact Sciences) | L3—Diagnostic thinking efficacy  L4—Therapeutic efficacy  L6—Societal outcome efficacy |
| 47 | McSorley et al., 2020 | Real-world analysis of clinical and economic impact of 21-gene recurrence score (RS) testing in early-stage breast cancer (ESBC) in Ireland | Ireland | Retrospective | Breast | Oncotype Dx (Genomic Health/Exact Sciences) | L3—Diagnostic thinking efficacy  L4—Therapeutic efficacy  L6—Societal outcome efficacy |
| 48 | Meldi et al., 2016 | A prospective, multi-center study to evaluate the performance and clinical utility of a 15-gene expression profile for uveal melanoma | USA | Prospective | Uveal Melanoma | DecisionDx-UM (Castle Biosciences) | L3—Diagnostic thinking efficacy |
| 49 | Michalopoulos et al., 2014 | Influence of a genomic classifier on post-operative treatment decisions in high-risk prostate cancer patients: Results from the PRO-ACT study | USA | Prospective | Prostate | Decipher (Veracyte) | L3—Diagnostic thinking efficacy |
| **No.** | **Authors, Year** | **Title** | **Geographic Location (Country)** | **Study Design** | **Type of Cancer Assessed** | **Assay Studied*** | **Reported Outcomes Against FT Model Levels** |
| 50 | Michaud et al., 2016 | French prospective multi-center cohort on the decision impact assessment | France | Prospective | Breast | Oncotype Dx (Genomic Health/Exact Sciences) | L3—Diagnostic thinking efficacy  L6—Societal outcome efficacy |
| 51 | Ozmen et al., 2015 | Results of the Turkish prospective multi-center study utilizing the 21-gene Oncotype DX assay: Decision impact analysis | Turkey | Prospective | Breast | Oncotype Dx (Genomic Health/Exact Sciences) | L3—Diagnostic thinking efficacy |
| 52 | Ozmen et al., 2016 | Impact of Oncotype DX Recurrence Score on Treatment Decisions: Results of a Prospective Multicenter Study in Turkey. | Turkey | Prospective | Breast | Oncotype Dx (Genomic Health/Exact Sciences) | L3—Diagnostic thinking efficacy |
| 53 | Petrakova et al., 2019 | Decision impact of the 21-Gene Oncotype DX Recurrence Score Assay (R) in the Czech Republic on recommendations for adjuvant chemotherapy in estrogen receptor positive early stage breast cancer (ESBC) patients | Czech Republic | Prospective | Breast | Oncotype Dx (Genomic Health/Exact Sciences) | L3—Diagnostic thinking efficacy |
| 54 | Plasseraud et al., 2016 | Clinical performance and management outcomes with the decision Dx-UM gene expression profile test in a prospective multicenter study | USA | Prospective | Uveal Melanoma | DecisionDx-UM (Castle Biosciences) | L3—Diagnostic thinking efficacy |
| 55 | Raphael et al., 2022 | The Impact of Comprehensive Genomic Profiling (CGP) on the Decision-Making Process in the Treatment of ALK-Rearranged Advanced Non-Small Cell Lung Cancer (aNSCLC) After Failure of 2nd/3rd-Generation ALK Tyrosine Kinase Inhibitors (TKIs) | Israel | Prospective & Retrospective | Lung | FoundationOne (Foundation Medicine) | L3—Diagnostic thinking efficacy  L4—Therapeutic efficacy  L5—Patient outcome efficacy |
| 56 | Reinbolt et al., 2016 | Decision impact analysis of comprehensive genomic profiling (CGP) in advanced breast cancer: A prospective study | USA | Prospective | Breast | FoundationOne (Foundation Medicine) | L3—Diagnostic thinking efficacy  L4—Therapeutic efficacy |
| 57 | Rezai et al., 2011 | Impact of the recurrence score on adjuvant decision-making in ER-positive early breast cancer - Results of a large prospective multicentre decision impact study in node negative and node positive disease | Germany | Prospective | Breast | Oncotype Dx (Genomic Health/Exact Sciences) | L3—Diagnostic thinking efficacy  L4—Therapeutic efficacy  L5—Patient outcome efficacy |
| 58 | Rodriguez et al., 2017 | Impact of the Prosigna (PAM50) assay on adjuvant clinical decision making in patients with early stage breast cancer: Results of a prospective multicenter public program | Spain | Prospective | Breast | Prosigna (Veracyte) | L3—Diagnostic thinking efficacy |
| **No.** | **Authors, Year** | **Title** | **Geographic Location (Country)** | **Study Design** | **Type of Cancer Assessed** | **Assay Studied*** | **Reported Outcomes Against FT Model Levels** |
| 59 | Rouzier et al., 2016 | Prospective multicenter study of the impact of the Prosigna assay on adjuvant clinical decision-making in women with early stage breast cancer: Which patients are the best candidates? | France | Prospective | Breast | Prosigna (Veracyte) | L3—Diagnostic thinking efficacy |
| 60 | Russell et al., 2017 | Treatment choices based on multiplatform profiling platform, unlike those with sequencing alone, do not cause a cost explosion in refractory cancer patients | Australia | Prospective & Retrospective | Refractory | Caris Molecular Intelligence (Caris Life Sciences) | L3—Diagnostic thinking efficacy  L6—Societal outcome efficacy |
| 61 | Sanft et al., 2015 | Prospective assessment of the decision-making impact of the Breast Cancer Index in recommending extended adjuvant endocrine therapy for patients with early-stage ER-positive breast cancer | USA | Prospective | Breast | Breast Cancer Index (Biotheranostics) | L3—Diagnostic thinking efficacy  L4—Therapeutic efficacy  L5—Patient outcome efficacy |
| 62 | Sanft et al., 2019 | A prospective decision-impact study incorporating Breast Cancer Index into extended endocrine therapy decision-making | USA | Retrospective | Breast | Breast Cancer Index (Biotheranostics) | L3—Diagnostic thinking efficacy  L5—Patient outcome efficacy  L6—Societal outcome efficacy |
| 63 | Sanft et al., 2015 | Prospective study of the decision-making impact of the Breast Cancer Index in the selection of patients with ER plus breast cancer for extended endocrine therapy. | USA | Prospective | Breast | Breast Cancer Index (Biotheranostics) | L3—Diagnostic thinking efficacy  L5—Patient outcome efficacy |
| 64 | Sankaran et al., 2021 | CanAssist Breast Impacting Clinical Treatment Decisions in Early-Stage HR+ Breast Cancer Patients: Indian Scenario | India | Retrospective | Breast | CanAssist Breast (OncoStem) | L3—Diagnostic thinking efficacy |
| 65 | Sethi et al., 2022 | Percepta Genomic Sequencing Classifier and decision-making in patients with high-risk lung nodules: a decision impact study | USA | Prospective | Lung | Percepta (Veracyte) | L3—Diagnostic thinking efficacy |
| 66 | Sethi et al., 2021 | The impact of a genomic sequencing classifier (GSC) on clinical decision making in patients with a high-risk lung nodule | USA | Prospective | Lung | Percepta (Veracyte) | L3—Diagnostic thinking efficacy |
| 67 | Shivers et al., 2022 | Interim analysis of the PREDICT Registry: Changes in treatment recommendation for a biologic signature predictive of radiation therapy (RT) benefit in patients with DCIS | USA | Prospective | Breast | DCISionRT Test (PreludeDx) | L3—Diagnostic thinking efficacy |
| 68 | Smyth et al., 2015 | Economic impact of 21-gene recurrence score testing on early stage breast cancer in Ireland | Ireland | Retrospective | Breast | Oncotype Dx (Genomic Health/Exact Sciences) | L3—Diagnostic thinking efficacy  L4—Therapeutic efficacy  L6—Societal outcome efficacy |
| **No.** | **Authors, Year** | **Title** | **Geographic Location (Country)** | **Study Design** | **Type of Cancer Assessed** | **Assay Studied*** | **Reported Outcomes Against FT Model Levels** |
| 69 | Smyth et al., 2015 | Economic impact of 21-gene recurrence score testing on early-stage breast cancer in Ireland | Ireland | Retrospective | Breast | Oncotype Dx (Genomic Health/Exact Sciences) | L3—Diagnostic thinking efficacy  L4—Therapeutic efficacy  L6—Societal outcome efficacy |
| 70 | Tharmabala et al., 2021 | An analysis of the clinical and economic impact of the 21-gene recurrence score (RS) in invasive lobular early-stage breast cancer (ESBC) in Ireland | Ireland | Retrospective | Breast | Oncotype Dx (Genomic Health/Exact Sciences) | L3—Diagnostic thinking efficacy  L4—Therapeutic efficacy  L6—Societal outcome efficacy |
| 71 | Thomas et al., 2018 | Multi-institutional, prospective clinical utility study evaluating the impact of the 92-gene assay (CancerTYPE ID) on final diagnosis and treatment planning in patients with metastatic cancer with an unknown or unclear diagnosis | USA | Prospective | Unknown Cancer | CancerType ID (Biotheranostics) | L3—Diagnostic thinking efficacy  L4—Therapeutic efficacy |
| 72 | Thomas et al., 2016 | Molecular diagnosis with the 92-Gene Assay (92-GA) and decision-impact on treatment: Final results from a prospective, multi-disciplinary study | USA | Prospective | Unknown Cancer | CancerType ID (Biotheranostics) | L3—Diagnostic thinking efficacy  L4—Therapeutic efficacy |
| 73 | Torres et al., 2018 | Prospective Evaluation of the Impact of the 21-Gene Recurrence Score Assay on Adjuvant Treatment Decisions for Women with Node-Positive Breast Cancer in Ontario, Canada | Canada | Prospective | Breast | Oncotype Dx (Genomic Health/Exact Sciences) | L3—Diagnostic thinking efficacy  L4—Therapeutic efficacy  L5—Patient outcome efficacy |
| 74 | Tramonti et al., 2018 | Decision Impact of a 21-gene signature in early breast cancer: A natural experiment using routine data | Scotland | Prospective | Breast | Oncotype Dx (Genomic Health/Exact Sciences) | L3—Diagnostic thinking efficacy  L6—Societal outcome efficacy |
| 75 | Tribedi et al., 2019 | An audit of the role of PONDx in chemotherapy decision-making in the breast MDT | England | Retrospective | Breast | Oncotype Dx (Genomic Health/Exact Sciences) | L3—Diagnostic thinking efficacy  L4—Therapeutic efficacy |
| 76 | Van Wert et al., 2016 | Impact of a bronchial genomic classifier for lung cancer on reducing invasive procedure recommendations across variations in pulmonology practices | USA | Prospective | Lung | Percepta (Veracyte) | L3—Diagnostic thinking efficacy |
| 77 | Villarreal-Garza et al., 2020 | Change in therapeutic management after the EndoPredict assay in a prospective decision impact study of Mexican premenopausal breast cancer patients | Mexico | Prospective | Breast | Oncotype Dx (Genomic Health/Exact Sciences) | L3—Diagnostic thinking efficacy  L4—Therapeutic efficacy |
| **No.** | **Authors, Year** | **Title** | **Geographic Location (Country)** | **Study Design** | **Type of Cancer Assessed** | **Assay Studied*** | **Reported Outcomes Against FT Model Levels** |
| 78 | Villarreal-Garza et al., 2019 | Change in therapeutic management after EndoPredict assay in a prospective decision impact study of Mexican premenopausal patients | Mexico | Prospective | Breast | Oncotype Dx (Genomic Health/Exact Sciences) | L3—Diagnostic thinking efficacy  L4—Therapeutic efficacy |
| 79 | Watanabe et al., 2019 | SUNRISE-DI study: decision impact of the 12-gene recurrence score (12-R5) assay on adjuvant chemotherapy recommendation for stage II and IIIA/B colon cancer | Japan | Prospective | Colon | Oncotype Dx (Genomic Health/Exact Sciences) | L3—Diagnostic thinking efficacy  L4—Therapeutic efficacy |
| 80 | Wuerstlein et al., 2019 | Strong impact of MammaPrint and BluePrint on treatment decisions in luminal early breast cancer: results of the WSG-PRIMe study | Germany,  Austria,  Switzerland | Prospective | Breast | Mammaprint (Agendia) | L3—Diagnostic thinking efficacy  L4—Therapeutic efficacy  L5—Patient outcome efficacy |
| 81 | Wuerstlein et al., 2016 | The West German study group breast cancer intrinsic subtype study: A prospective multicenter decision impact study utilizing the Prosigna assay for adjuvant treatment decision-making in estrogen-receptor-positive, HER2-negative early-stage breast cancer | Germany | Prospective | Breast | Prosigna (Veracyte) | L3—Diagnostic thinking efficacy  L5—Patient outcome efficacy |
| 82 | Wuerstlein et al., 2017 | Results of multigene assay (MammaPrint (R)) and molecular subtyping (BluePrint (R)) substantially impact treatment decision making in early breast cancer: Final analysis of the WSG PRIME decision impact study | Germany | Prospective | Breast | Mammaprint (Agendia) | L3—Diagnostic thinking efficacy  L4—Therapeutic efficacy |
| 83 | Wuerstlein et al., 2015 | Significance of prospective multicenter decision impact WSG-BCIST Study in post-menopausal ER+ HER2-N0 early breast cancer (EBC) for molecular testing for intrinsic subtype definition | Germany | Prospective | Breast | Prosigna (Veracyte) | L3—Diagnostic thinking efficacy  L4—Therapeutic efficacy |
| 84 | Yamauchi et al., 2014 | Prospective study of the effect of the 21-gene assay on adjuvant clinical decision-making in Japanese women with estrogen receptor-positive, node-negative, and node-positive breast cancer | Japan | Prospective | Breast | Oncotype Dx (Genomic Health/Exact Sciences) | L3—Diagnostic thinking efficacy  L5—Patient outcome efficacy |
| 85 | Yamauchi et al., 2012 | Societal economics of the 21-gene Recurrence Score in estrogen-receptor-positive early-stage breast cancer in Japan | Japan | Retrospective | Breast | Oncotype Dx (Genomic Health/Exact Sciences) | L3—Diagnostic thinking efficacy  L4—Therapeutic efficacy  L5—Patient outcome efficacy  L6—Societal outcome efficacy |
| **No.** | **Authors, Year** | **Title** | **Geographic Location (Country)** | **Study Design** | **Type of Cancer Assessed** | **Assay Studied*** | **Reported Outcomes Against FT Model Levels** |
| 86 | Yamauchi et al., 2011 | Decision Impact and Economic Evaluation of the 21-gene Recurrence Score (RS) Assay for Physicians and Patients in Japan | Japan | Prospective | Breast | Oncotype Dx (Genomic Health/Exact Sciences) | L3—Diagnostic thinking efficacy  L4—Therapeutic efficacy  L5—Patient outcome efficacy  L6—Societal outcome efficacy |
| 87 | Zambelli et al., 2020 | Prospective observational study on the impact of the 21-gene assay on treatment decisions and resources optimization in breast cancer patients in Lombardy: The BONDX study | Italy | Prospective | Breast | Oncotype Dx (Genomic Health/Exact Sciences) | L3—Diagnostic thinking efficacy  L4—Therapeutic efficacy  L6—Societal outcome efficacy |

*We list the current company name associated with each assay (and not necessarily the company’s name associated with each assay at the time when the item was published).
