# Supplementary material for: Decision impact studies, evidence of clinical utility for genomic assays in cancer: A scoping review
Source: PLoS One. 2023 Mar 10;18(3):e0280582. doi: 10.1371/journal.pone.0280582 (PMC10004522; doi:10.1371/journal.pone.0280582)
Supplement: Attachment — Submitted filename: Plos One - Parker et al. - Response Letter - Oct2022.docx [file pone.0280582.s004.docx]

Dear Dr. De Mello,

Thank you for the invitation to revise and resubmit our manuscript. We have addressed the reviewer’s comments and believe that incorporating their feedback has enhanced the quality of our paper. Their comments were insightful, and we believe this review process has resulted in an improved manuscript. A detailed response to the comments may be found in the table below.

Sincerely,

Gillian Parker

|  | **Reviewer Comment** | **Action** |
| --- | --- | --- |
| **Title** | | |
| *1* | *Please try to express in as few words as possible.* | We have edited the title. |
| **Abstract** | | |
| *2a* | *Try to synthetize the background section. Extend methods, you can briefly describe the inclusion and exclusion criteria using in the study selection and define your variables. How to categorize the types of clinical utility outcomes reported?* | Thank you for this suggestion. We have edited and synthesized the abstract. |
| *2b* | *Try to select only three or four keywords.* | We have reduced the key words to six. |
| **Background** | | |
| *3* | *There are six paragraphs, please* ***try to resume in four paragraphs****.*  *The first paragraph is good; in this you explain the utility of DIS. The second paragraph is regarding the use of genomic assays in cancer care. The third and the fourth paragraph could be* ***summarized in only one paragraph****.* ***You should focus on the importance to assess clinical utility and the most accepted definition****. Finally, the fifth and the sixth paragraph* ***could be only one****,* ***try to explain the motivation for doing that research, show the knowledge gap that will lead into the importance of your study.*** | Thank you for this suggestion. We have edited and re-organized the background section. |
| **Study Objectives** | | |
| *4* | *You repeat the importance of DIS. If the primary outcome is the impact of a genomic assay on treatment decisions or recommendations for a specific population of cancer patients.* ***I am not sure if the primary objective should be only identify and characterize decision impact studies published for genomic assays in cancer care.*** *Maybe you can change the primary objective for the second: describe the types of clinical utility outcomes reported as evidence of DIS in clinical and reimbursement decision-making.* | Thank you for this observation. We have removed the duplicated statement. We decided to conduct a scoping review because there is no published synthesis of the literature on decision impact studies. A scoping review is foundational study to establish the nature and scope of a body of research.  We have clarified this objective in the manuscript. |
| **Methods** | | |
| *5a* | *What type of studies were included?* | Thank you for identifying this omission. We have added detail regarding ‘type of studies’ included in this review. |
| *5b* | *How many meta- analyses were conducted on that topic? Has the literature evolved significantly to justify an up- to- date meta- analysis? Are there any relevant studies missing in the already published meta- analyses?* | Thank you for this inquiry. While two meta-analyzes on tangentially related topics (comparing radiomic MRI and RS (2021) and use of Oncotype Dx (2015)) have been published and cite some DIS studies in their included studies, to our knowledge, no reviews have been conducted of decision impact studies. This fact motivated us to conduct this review. Our intention is to provide a comprehensive synthesis of DIS and contribute to foundational knowledge in this emerging field of research.  We have explicitly stated the gap in knowledge synthesis in this field in the objectives statement. |

|  | **Addition changes** |  |
| --- | --- | --- |
| *6* | Change Corresponding Author | Please note the corresponding author has been changed in the manuscript – from Gillian Parker to Fiona A. Miller.  Additionally, we are requesting that this change be made in the online system for correspondence. |
